# Metabolomic profiling of cannabis use and cannabis intoxication in humans

**DOI:** 10.1038/s41386-025-02082-7

**Published:** 2025-03-12

**Authors:** Francisco Madrid-Gambin, Noemí Haro, Natasha L. Mason, Pablo Mallaroni, Eef L. Theunissen, Stefan W. Toennes, Oscar J. Pozo, Johannes G. Ramaekers

**Affiliations:** 1 Applied Metabolomics Research Group, Hospital del Mar Research Institute, 08003 Barcelona, Spain; 2 Department of Neuropsychology and Psychopharmacology, Faculty of Psychology and Neuroscience, Maastricht University, 6200 MD Maastricht, the Netherlands; 3 Goethe University Frankfurt, University Hospital, Institute of Legal Medicine, Frankfurt, Germany

**Keywords:** Neuroscience, Psychology

## Abstract

Acute intoxication from Δ9-tetrahydrocannabinol (THC, the primary active ingredient of cannabis) can lead to neurocognitive impairment and interference with day-to-day operations, such as driving. Present evaluations of THC-induced impairment in legal settings rely on biological drug tests that solely establish cannabis use, rather than cannabis impairment. The current study evaluated the metabolome in blood collected from occasional and chronic cannabis users (*N* = 35) at baseline and following treatments with cannabis (300 μg/kg THC) and placebo, with the aim to identify unique metabolic alterations that are associated with acute cannabis intoxication and cannabis use frequency. Blood samples were collected at baseline and repeatedly during 70 min after treatment. Sustained attention performance and ratings of subjective high were taken twice within 40 min after treatment. Metabolomic fingerprints of occasional and chronic cannabis users were distinctly different at baseline, when both groups were not intoxicated. A total of 14 metabolites, mainly related to endocannabinoid and amino acid metabolism, were identified that distinguished chronic from occasional cannabis users and that yielded a discriminant analysis model with an 80% classification rate (95% CI: 61–91%). Distinct metabolomic fingerprints were found for occasional cannabis users who, in contrast to chronic cannabis users, showed attentional impairment and elevated ratings of subjective high during cannabis intoxication. These included increments in organic acids, β-hydroxybutyrate and second messenger ceramides. The current study demonstrates the feasibility of the metabolomics approach to identify metabolic changes that are specific to the neurocognitive state of cannabis intoxication and to the history of cannabis use.

## Introduction

Cannabis belongs to the most used psychoactive substances worldwide [1]. Prevalence data from 2020 suggests that more than 4% of the global population aged 15-64 (209 million people) used cannabis in the past year [2]. A major concern raised about the use of cannabis is that it might induce neurocognitive deficits, e.g. impairments of cognitive and motor function, during acute intoxication [3] or following long-term use [4–6], that should be monitored or sanctioned when cannabis users engage in safety-sensitive operations. This concern has gained even more traction with the increasing interest in medical applications of cannabis [7–9].

Acute consumption of delta-9-tetrahydrocannabinol (THC), i.e., the prime active component in cannabis, has been associated with transient impairments of cognitive and psychomotor function in occasional cannabis users, while its impact on neurocognition appears less prominent or even absent in chronic users due to tolerance [10–12]. Temporal behavioral changes to acute cannabis exposure have been attributed to neuroadaptations, such as CB1 receptor downregulation, and altered dopaminergic and glutamatergic neurotransmission in the brain that develop over the time course of frequent cannabis use [3, 10]. Yet, individual variations in the neurocognitive effects of cannabis are often overlooked when assessing and defining levels of intoxication in legal contexts, such as driving under the influence of cannabis [13]. Currently, there is no reliable method to differentiate between cannabis use that leads to impairment and use without impairment [14]. The standard procedure for assessing acute cannabis intoxication involves drug tests to assess THC in bodily fluids such as oral fluid and blood. However, the presence of THC in biological samples is not a strong indicator of cannabis-induced impairment [3, 15, 16]. Hence there is a strong quest to date to develop a predictive parameter that can be used to selectively and reliably determine cannabis-induced impairment, rather than cannabis use [14, 17].

The physiological mechanism and molecular pathways involved in the temporal response to acute cannabis intoxication have been poorly researched in humans. In this regard, the use of metabolomics may provide a functional readout of the physiological state of cannabis users following acute and repeated cannabis exposure. Metabolomics is the study of chemical processes involving metabolites and as such can be used to determine unique chemical fingerprints left behind by cellular processes [18]. Evaluation of dysregulated sections of the metabolome of a particular biospecimen can elucidate insights into biological and physiological processes in psychopathological states [19] as well as in states of drug intoxication [20], including cannabis, as shown in preclinical studies [21]. These findings provide support for the current objective to utilize metabolomics for the identification of unique alterations in the blood metabolome that are associated with cannabis intoxication and cannabis use frequency. Establishing such a metabolomic fingerprint may allow for the ability to classify and separate cannabis use and cannabis impairment, an endeavor that may have important forensic applications [3]. In the current study, we applied a targeted LC–MS/MS metabolomics approach in a placebo-controlled study with occasional and chronic users of cannabis to identify the metabolic signature of cannabis use and the neurocognitive state of cannabis intoxication in blood.

## Materials and methods

### Subjects and study design

Thirty-five healthy cannabis users with a mean (SD) age of 22.0 ( ± 2.2) years were enrolled in a double‐blind, placebo‐controlled, mixed cross‐over design in cannabis users. Inclusion criteria were 18–40 years; body mass index between 18 and 28 kg/m2; and written informed consent. Exclusion criteria were history of drug abuse (other than the use of cannabis) or addiction; pregnancy or lactation; health issues including hypertension (diastolic > 90 and systolic > 140), cardiac dysfunction, and liver dysfunction; current or history of endocrine, neurological, psychiatric disorders; and use of psychotropic medication.

Each participant inhaled cannabis-placebo and cannabis (300 μg/kg THC) by vaporizer on separate days, separated by a minimum wash‐out period of 7 days. Order of treatments were counterbalanced across participants. Medical cannabis (Bedrobinol; 13.5% THC) was obtained from the Office of Medicinal Cannabis, the Netherlands. Treatment orders were randomly assigned to participants. Participants were classified into occasional (*N* = 18) and chronic (*N* = 17) users of cannabis based on their cannabis use history. Occasional cannabis users were defined as individuals using cannabis up to 3 times a week for the past year, while chronic cannabis users consumed cannabis at least 4 times a week for the past year. Previous studies have demonstrated that this user group classification, at the same cannabis dose as selected in the present study, allows differentiation between cannabis users who are non-tolerant or (partial) tolerant to an acute challenge with cannabis [22, 23]. In case of occasional cannabis users, cannabis or cannabis-placebo was only administered if a subject had passed the alcohol and drug screens on a given test day. In case of a positive drug screen, occasional users were sent home to return to the laboratory at a later time. In case of chronic cannabis users, cannabis and cannabis-placebo were administered if subjects tested positive for THC, but negative for other drugs and alcohol. Chronic cannabis users always tested positive for THC on test days, prior to dosing.

Participant demographics (age, sex, cannabis use) of occasional and chronic cannabis users are shown in Table S1. The study was conducted at the Faculty of Psychology and Neuroscience at Maastricht University in accordance with the code of ethics on human experimentation established by the declaration of Helsinki (1964), and amended in Fortaleza (Brazil, October 2013) and approved by the medical ethical review board of the Academic Hospital Maastricht and Maastricht University. This study was registered in the Netherlands Trials Register (NL-OMON27947). A permit for obtaining, storing, and administering cannabis was obtained from the Dutch Drug Enforcement Administration. Data collected in this study consists of metabolomics that were conducted on serum blood samples collected at baseline and at 10, 30, 50 and 70 min after treatment and of cognitive and subjective high measures that were taken at 10 and 30 min after treatment and at 20 and 40 min after treatment, respectively. Measures of cognition and subjective high were taken during the time window where effects of THC were expected to be maximal [23, 24]. Serum samples were extracted, aliquoted, encoded, and frozen at −20 °C until they were used.

### Cognitive and subjective measures

Attentional performance was assessed with a psychomotor vigilance task (PVT), a 6-min reaction‐time task that measures the speed with which participants respond to a visual stimulus. The main measure of the task is the number of attentional lapses (reaction time > 500 ms). The task is a gold standard in sleep research to assess sustained attention or vigilance and has been applied to monitor an individual’s performance capacity at the workplace [25]. We focused on a single performance measure because acute THC effects on multiple performance domains (e.g. attention, psychomotor function, memory and decision making) rarely occur in isolation but collectively appear across all of these domains as these are all rooted in same underlying neural circuit, i.e. the mesocorticolimbic circuit [3]. We therefore selected a performance measure that is very sensitive to the effect of THC on the mesocorticolimbic circuit [12, 22] with the expectation that this effect will also generalize to other performance domains. Participants also rated their subjective high on a visual analog scale between 0 (not high at all) and 10 (extremely high).

### Metabolomics profiling and cannabinoid determination

After protein precipitation, five protocols were followed for each family of compounds (carboxylic acids, polar neurotransmitters, lipids, tryptophan and tyrosine metabolism, and steroids) using previously reported methods [26–31]. A set of 91 targeted biomarkers and 22 ratios (Table S2) were determined by selected reaction monitoring by a liquid chromatography coupled to tandem mass spectrometry (LC–MS/MS) system consisting of an Acquity UPLC instrument (Waters Associates, Milford, MA, USA) coupled to a triple quadrupole (TQS Micro, Waters) mass spectrometer. MassLynx software V4.1 (Waters Associates) was used for peak integration and data management. Cannabinoids (THC, 11-hydroxy-THC and 11-nor-9-carboxy-THC) were analyzed as described previously [32].

### Statistical analysis

Data was imported to R software version 4.2 for statistical analyses [33]. To evaluate demographic data between cannabis user groups, Pearson’s Chi-squared test and independent Student’s t test were applied on categorical and numerical data, respectively. Before performing principal component analysis (PCA) on metabolomic data sets, missing values were imputed using the Bayesian PCA algorithm [34] on log-transformed Pareto-scaled data.

Baseline metabolic differences between chronic and occasional cannabis smokers were assessed using a repeated double cross-validation partial least squares discriminant analysis (rdCV-PLS-DA) with feature selection based on recursive ranking informed by Variable Importance in Projection [35]. The significance of the model was evaluated through a permutation test with 200 iterations, where randomly permuted class labels were compared against the actual model.

To analyze time-series of metabolic changes in chronic and occasional users after vaporization of cannabis, we utilized generalized linear mixed model across the time points [36] in each user group. Treatment (2 levels: cannabis, placebo), Time (5 levels: 0, 10, 30, 50 and 70 min) and Treatment by Time interaction were used as fixed effects, baseline value as covariate and subject as random factor, and individual within-subject treatment as the repeating unit with a 1st order autoregressive covariance structure. In case of significant treatment × time interactions, treatment effects per time point were examined.

Change scores (Δ-data) were calculated to associate metabolomic data with attentional performance and subjective high. The Δ-lapses in attention were computed by subtracting individual performance scores in the placebo condition from those in the cannabis condition, at two distinct time points, namely 10- and 30-min post-vaporization. Similarly, Δ-subjective effects were determined by subtracting subjective high scores in the placebo condition from those in the cannabis condition. Subsequently, the metabolic changes associated with the Δ-lapses of attention and Δ-subjective high were analyzed by subtracting baseline values from the metabolomics data obtained at 10 min and 30 min (designated as Δ-metabolomics 10 min and Δ-metabolomics 30 min). The Δ-lapses of attention served as the dependent variable in generalized linear models, with the Δ-metabolomics data, age, sex, and baseline THC concentrations as independent predictors, stratified by user type and timepoint. Similarly, Δ-subjective high ratings were evaluated using the same approach.

Hierarchical family-wise false discovery rate (FDR) was applied to account for multiple comparisons [37, 38]. An FDR-adjusted *p* value < 0.05 was considered statistically significant.

## Results

Mean (SD) THC concentrations at 10 and 30 min after THC vaporization were 9.69 (6.01) and 3.41 (2.03) ng/ml in occasional cannabis users and 17.10 (14.2) and 7.63 (4.66) ng/ml in chronic users. Chronic cannabis users also tested positive for residual THC at baseline, revealing a mean (SD) THC concentration of 2.99 (2.42). Occasional users were negative for THC at baseline.

### Baseline metabolic differences between chronic and occasional smokers of cannabis

**Fig. 1:**
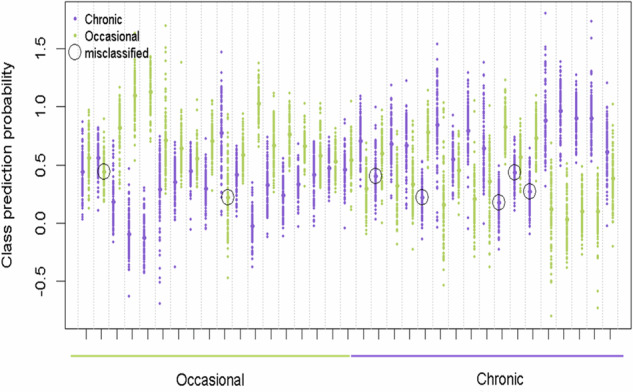
Misclassification plot from a repeated double cross-validated Partial Least Squares Discriminant Analysis model. The misclassification plot shows each sample predicted as “occasional” and “chronic” users. The first half of the horizontal axis represents actual occasional users, while the second half represents actual “chronic” users. Bold points are the average predicted samples, while other points display predictions from each iteration. Misclassifications are differences between predicted user group compared to the actual user group, as shown by black circles.

The rdCV-PLS-DA analysis yielded a model achieving an 80% classification rate, with a 95% confidence interval ranging from 63 to 91%, supported by a permutation test *p* value of <0.01 (see Fig. 1). Fourteen discriminant metabolites were identified through multivariate modeling, as detailed in Table 1. Notably, the metabolic profile associated with chronic cannabis users predominantly implicated pathways related to endocannabinoid and amino acid metabolism. Specifically, chronic users exhibited lower circulating levels of 2-acyl-glycerol-based endocannabinoids, including 2-arachidonoyl glycerol (2-AG) and 2-oleoylglycerol (2-OG), as well as lower concentrations of metabolites stemming from aromatic amino acids metabolism such as tyrosine and phenylalanine. Additionally, lower levels of metabolites from the kynurenine pathway, namely kynurenine, kynurenic acid, and 5-hydroxyindoleacetic acid (5HIAA), along with diminished concentrations of branched-chain amino acids including valine and leucine, were observed in chronic users. Levels of ceramide18:1 14:0 and hexanoic acid were higher in chronic users as compared to occasional users.

**Table 1:** Baseline metabolomic signature (mean ± SD) that discriminates between chronic and occasional cannabis users (ng/mL) based on a rdCV-PLS-DA model.

Metabolite	Family	Chronic	Occasional	Order LR^a^	Rank LR
Tyrosine	AAA metabolism	9120.561 ± 2380.824	11893.329 ± 2150.54	1	4.9
Phenylalanine	AAA metabolism	8691.477 ± 1702.35	10246.299 ± 1089.711	2	13.8
Cer(d18:1/14:0)	Ceramide	17.391 ± 7.164	12.825 ± 4.917	3	21.2
Kynurenine	TRP metabolism	298.725 ± 56.794	365.111 ± 75.766	4	22.2
Kynurenic acid	TRP metabolism	6.663 ± 2.064	8.999 ± 2.663	5	24.9
2-AG	Endocannabinoid	0.553 ± 0.319	0.744 ± 0.219	6	35
2-OG	Endocannabinoid	3.642 ± 1.613	4.626 ± 1.827	7	35.7
TMAO	Amine oxide	28.396 ± 16.148	48.527 ± 30.33	8	41.4
Creatine	Protein metabolism	1727.957 ± 1263.148	2959.971 ± 1702.267	9	45.8
Valine	BCAA metabolism	20920.237 ± 8692.164	30634.526 ± 12981.122	10	48.4
5HIAA	TRP metabolism	2.55 ± 1.935	4.241 ± 3.799	11	52
Leucine	BCAA metabolism	16703.03 ± 4198.31	19634.586 ± 4386.404	12	55.1
Hexanoic acid	Saturated fatty acid	9.299 ± 3.548	7.151 ± 2.164	13	55.6
5b-THF	Steroid	0.466 ± 0.254	0.732 ± 0.448	14	56.8

*2-AG* 2-arachidonoyl glycerol, *2-OG* 2-oleoylglycerol, *5b-THF* 5β-tetrahydrocortisol, *5HIAA* 5-hydroxyindoleactic acid, *AAA* aromatic amino acid, *BCAA* branched-chain amino acid, *TMAO* trimethylamine N-oxide, *TRP* tryptophan.

^a^Loading rank of rdCV-PLS-DA.

### Metabolic changes after vaporization of cannabis

**Fig. 2:**
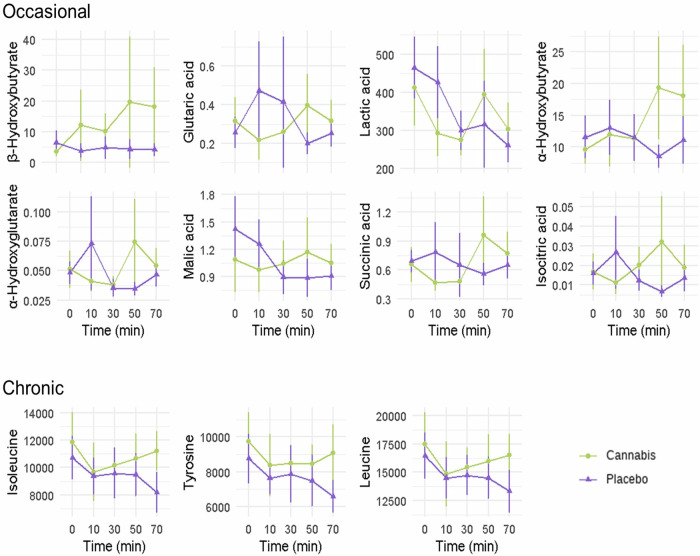
Metabolic changes during cannabis intoxication. Kinetics of significant metabolites, expressed as mean concentrations (ng/mL) with confidence intervals from modeling, in occasional and chronic users during cannabis intoxication, compared to placebo (only metabolites with FDR-adjusted *p* values < 0.05 are shown).

Table S3 presents full outcomes from the time-series analysis on baseline-corrected metabolomics in occasional and chronic users after vaporization of cannabis, as compared to placebo. In occasional users, vaporization of cannabis increased metabolite concentrations of β-Hydroxybutyrate (W = 7.44; *p* = 0.033), lactic acid (W = 6.44;p = 0.033), malic acid (W = 5.14; *p* = 0.047), succinic acid (W = 4.87; *p* = 0.047), isocitric acid (W = 4.69; *p* = 0.047), glutaric acid (W = 7.41; *p* = 0.033), α-hydroxybutyrate (W = 6.43; *p* = 0.040), α-hydroxyglutarate (2-hydroxyglutarate) (W = 5.73; *p* = 0.047) (Fig. 2, for comparative data of chronic users see Fig. S1). Concentrations of β-hydroxybutyrate were most notably elevated relative to placebo. Trend towards sustained reductions in N-palmitoylethanolamine (PEA) and docosatetraenoylethanolamide (DEA) after use were observed after cannabis vaporization, but these did not survive FDR correction for multiple comparisons.

In chronic users, concentrations of the isoleucine (W = 9.90; *p* = 0.038), leucine (W = 7.87; *p* = 0.039) and tyrosine (W = 8.13; *p* = 0.039) increased after acute cannabis vaporization, as compared to placebo (Fig. 2, for comparative data of occasional users see Fig S1). These increments appeared most evident around 50–70 min after vaporization. Sustained elevations of cortexolone and beta-hydroxybutyrate concentrations were also observed, but these did not pass FDR correction.

### Metabolomics and attention

**Fig. 3:**
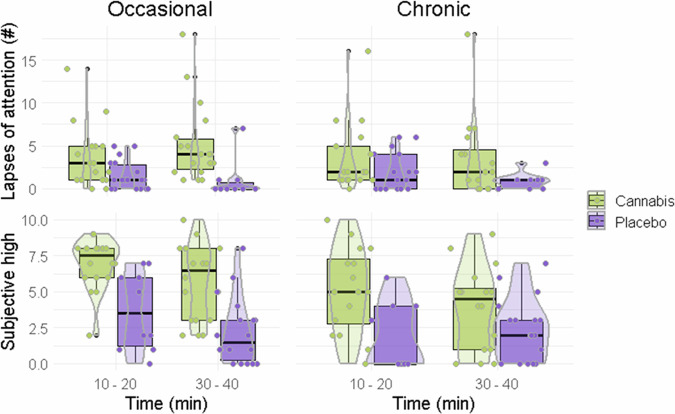
Attentional performance and subjective high. Boxplots of attentional performance (lapses of attention) and ratings of subjective high as a function of time after cannabis and placebo treatment in occasional and chronic cannabis users.

Mixed linear models revealed significant effects of Treatment (F_1,31.66_ = 5.338; *p* = 0.028) and Time(F _1,38.35_ = 7.351; *p* = 0.01) on attentional performance in occasional cannabis users, but not in chronic users. Post hoc power analyses revealed a power of 53% to detect effects of Treatment and Time respectively in occasional users. These findings indicate that the mean number of lapses of attention increased under cannabis in occasional users but was unaffected in chronic users, as shown in Fig. 3.

**Fig. 4:**
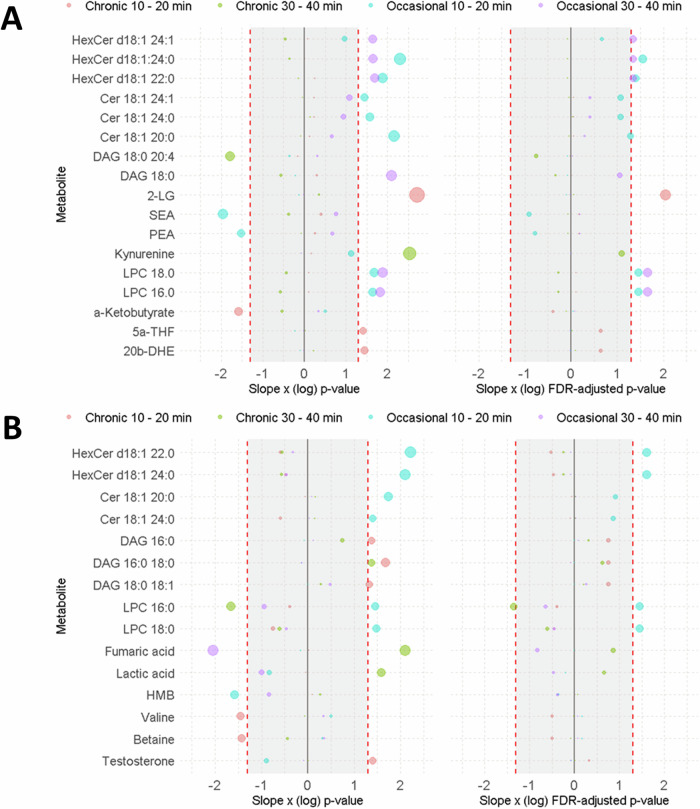
Metabolites associated with lapses of attention and subjective high. A summary of changes in metabolites that significantly (i.e., below or above the gray demarcation zone) associated with changes in attentional performance (**A**) and subjective high (**B**) in both cannabis user groups at 2 time points after cannabis administration. The left panel shows uncorrected *p* values, the right panel shows FDR corrected *p* values.

In occasional users, a significant association emerged between change in lapses of attention at 10 min post-cannabis and specific lipids (shown in Fig. 4 and Table S4), predominantly hexosylceramides (HexCer d18:1.22:0 and HexCer d18:1.24:0), ceramides (Cer18:1 20:0) and lysophosphatidylcholines (LPC 16:0 and LPC 18:0). At 30 min post-cannabis, the association with LPCs and attention task performance persisted. The lipids HexCer d18:1.22:0, HexCer d18:1.24:0, and HexCer d18:1.24:1 also yielded associations with changes in attention task performance at 30 min post-cannabis. In chronic users, only 2-linoleoylglycerol (2-LG) exhibited a positive association with changes in lapses of attention at 10 min post-cannabis, as shown in Fig. 4A and Table S5. Notably, there were no significant associations at 30 min post-cannabis.

### Metabolomics and subjective high

Mixed linear models revealed a significant effect of Treatment (F_1, 34.79_ = 72.63, *p* < 0.001) and Time (F_1, 52.57_ = 5.12; *p* = 0.028) on subjective ratings of high in occasional cannabis users as well as in in chronic users (Treatment: F_1,35.61_ = 35.61; *p* = <0.001; Time: F_1,51.93_ = 8.08; *p* = 0.006). Post hoc power analyses revealed a power of 99% and 52% to detect effects of Treatment and Time respectively in occasional users, and a power of 99% and 51% respectively in chronic users. These findings indicate that mean ratings of subjective high increased under cannabis in both cannabis user groups as depicted in Fig. 3.

The associations between metabolic markers and subjective effects were limited. A positive association was observed with lipids HexCer.d18:1 22:0, HexCer.d18:1 24:0, LPC 16:0 and LPC 18:0 at 20 min post cannabis among occasional users (see Fig. 4B and Table S6). No significant associations were found between subjective effects and metabolic markers at 40 min post cannabis among occasional users (see Fig. 4B and Table S7). With the exception of LPC 16:0, no significant associations were observed between metabolic markers and subjective effects in chronic users.

## Discussion

The present study aimed to identify metabolomic profiles of occasional and chronic users of cannabis, at baseline and after vaporization of cannabis and placebo. We identified significant variations in metabolism between these groups at baseline and following cannabis intoxication. Additionally, we observed metabolic changes associated with the degree of subjective intoxication and the neurocognitive state of cannabis intoxication.

A primary finding of the present study is that metabolomic fingerprints of occasional and chronic cannabis were markedly different at baseline, when both groups were not intoxicated. A total of 14 discriminant metabolites were identified that distinguished chronic from occasional cannabis users. Overall, chronic cannabis users showed lower concentrations of metabolites resulting from aromatic amino acid metabolism (e.g. tyrosine, phenylalanine), including tryptophan metabolism (kynurenine, kynurenine acid, 5-HIAA), and branched-chain amino acid metabolism (e.g. valine, leucine). In addition, chronic cannabis users displayed reduced levels of endocannabinoid related metabolites such as 2-AG and 2-OG and elevated levels of ceramides. Reductions in amino acid metabolism and endocannabinoids might be related to prolonged neuroadaptations that have been described in chronic cannabis users, such as CB1 receptor downregulation [10, 39]. Downregulation of CB1 receptors can reduce the functional role of CB1 receptors in the synthesis of endocannabinoids [40, 41] and monoamine metabolites [42, 43] and alter the interconnection of cannabinoid and monoamine signaling pathways [43]. CB1 receptors are also coupled to the generation of the lipid second messenger ceramide [44, 45]. Long-term accumulation of ceramide, as observed in the present study, has previously been reported after prolonged CB1 receptor activation in preclinical studies and has been suggested to mediate cannabinoid-induced apoptosis in tumor cells [44, 46]. The metabolomic fingerprint of chronic cannabis users thus appears in line with the current literature on the impact of long-term cannabis use on metabolic pathways. Discriminant analysis model classifications of participants into occasional or chronic users achieved an 80% accuracy rate, suggesting a consistent level of distinction between the metabolomic profiles of occasional and chronic cannabis users. If validated in other populations, metabolic fingerprints of chronic and occasional cannabis users might potentially serve to identify cannabis use history in individual users.

Metabolomic profiles of cannabis intoxication also differed between occasional and chronic cannabis users. In occasional users, acute exposure to cannabis significantly increased a group of organic acids involved in biochemical reactions, such as the citric acid cycle (also known as Krebs cycle), that release energy stored in nutrients to support cellular bioenergetics [47]. Similarly, increments in ketone body concentrations such as β-hydroxybutyrate were observed that may reflect increased energy metabolism, as the latter is converted to acetyl-Coenzyme A (acetyl-CoA) which enters the citric acid cycle to be oxidized for energy [48]. Interestingly, release of ketone bodies such as β-hydroxybutyrate has been implicated in the synthesis and homeostasis of brain concentrations of glutamate and GABA [49]. This may be in line with previous observations of increased striatal glutamate concentration in occasional users, but not chronic users, after a single exposure to cannabis [22, 50]. Cannabis-induced changes in β-hydroxybutyrate were also most consistent in occasional users as elevations were observed throughout the entire interval between 10 and 70 min after vaporization.

In chronic users, cannabis-induced changes in the metabolomic profile were limited to elevations in three amino acids, i.e., isoleucine, leucine and tyrosine, which have been implicated in protein synthesis and cell function [51]. However, functions of these amino acids in systems biology are multiple and diverse. For instance, tyrosine is known as a precursor of the neurotransmitter dopamine [52, 53], while isoleucine and leucine transamination may contribute to glutamate synthesis [54, 55]. These findings seem in line with a previous metabolomics study in cannabis users, which reported that levels of the amino acid taurine in urine increased with increasing cannabis use [56]. However, the mechanisms underlying the differential metabolomic profiles of occasional and chronic cannabis users when exposed to cannabis are currently unknown. They might be related to the development of behavioral and physiological tolerance to acute effects of cannabis that has been well documented [10, 11]. Repeated exposure to cannabis has been modeled to lower the release of dopamine and glutamate in the mesocorticolimbic circuit in chronic users through CB1 receptor downregulation [3, 57]. It might be speculated therefore, that increments in amino acid concentrations following cannabis exposure in chronic users might serve to increase resources from which dopamine and glutamate might be synthesized in order to regain their homeostasis.

Metabolomic fingerprints could also be associated to behavioral changes recorded during cannabis intoxication. Behavioral measures included subjective rating of high and an objective measure of attentional performance. Subjective ratings of high were significantly increased after cannabis vaporization in both cannabis user groups, whereas attentional deficits were observed after cannabis vaporization in occasional users but not in chronic users. Cannabis induced changes in attention and subjective high were significantly associated with increments in (hexosyl)ceramides in occasional users. Ceramides are second messengers present in low concentrations in resting cell-surface and can be rapidly produced or released through CB1 receptor-evoked sphingomyelin hydrolysis [45, 58]. Sustained ceramide accumulation through CB1 activation has been implicated in regulating apoptosis (programmed cell death) and cellular proliferation [45, 59]. Interestingly, plasma ceramide concentrations have been suggested as potential early blood-based biomarkers for predicting mild cognitive impairment and progression to dementia [60] as studies have reported an association between higher plasma ceramide concentrations and hippocampal atrophy and cognitive decline [61]. Such findings appear in line with cannabis-induced impairment of cognitive function as observed in the present study.

Cannabis-induced changes in attention were also associated with increments in LPC 16:0 and 18:0 and, though not significantly after FDR correction, DAG 18:0 in occasional cannabis users. Additionally, cannabis-induced changes in LPC also associated with changes in subjective high. LPC is a lipid mediator with pro-inflammatory activities [62]. DAG 18:0 is involved in the production of 2-LG that acts as a partial agonist of CB1 receptors [63]. Moreover, 2-LG  has also been shown to blunt the activity of endocannabinoids such as 2-Arachidonolyglycerol (2-AG) and anandamide (AEA) [63]. It is interesting in this regard that 2-LG was also the only metabolite that was significantly associated to attentional performance in chronic cannabis users, even when mean attentional performance in this group was not significantly affected under cannabis. The current finding suggests that increased levels of 2-LG following cannabis use may contribute to a blunted cognitive response in chronic cannabis users by active suppression of endocannabinoid activity. Our finding that baseline endocannabinoid levels of 2-AG and 2-OG were lowered in chronic users further underscores this notion.

The current placebo-controlled study is the first to comprehensively assess metabolomic profiles of individuals who have a history of cannabis use during active cannabis intoxication and baseline and the first to provide scientific evidence to the notion that cannabis effects on neurocognition can be predicted from its impact on the metabolome. The present findings provide a proof of the principle that changes in lipid metabolism, energy metabolism, and neurotransmitter pathways that are specific to the neurocognitive state of cannabis intoxication and to frequency of cannabis use can be determined in blood. In principle, the metabolomics approach might serve to identify specific biomarkers that are consistently associated with cannabis use frequency and cannabis intoxication and might allow differentiation of a behavioral response of individuals to an acute cannabis challenge. Such an approach might offer confirmatory and complementary information to current biomarkers of cannabis consumption, such as THC or its metabolites, as these can only be used to determine recent or past use of cannabis, but not the neurocognitive state of intoxication [3]. Giving changing legality and day to day use of cannabis, metabolomic biomarkers can help inform legal instances where it is relevant to separate cannabis use from cannabis impairment, such as in cases of driving under the influence of cannabis.

It should be noted however, that though the current study offers an innovative path to the development of a metabolomics fingerprint that is predictive of cannabis impairment, it does not yet provide a definitive test. In order to move forward, the reliability and sensitivity of the metabolomics approach to identify behavioral changes in cannabis users during intoxication should be replicated in larger samples and be further developed to allow metabolomic fingerprints of single blood samples, in the absence of a baseline reference. Alternatively, acquiring a baseline (non-intoxicated) sample at some timepoint after recovery from cannabis intoxication could also have utility in ‘real world’ settings in the absence of a pre-use baseline. Future investigations on the association between cannabis induced changes in metabolomics and psychomotor vigilance performance should also track the full pharmacodynamic time course of cannabis intoxication, and determine the specificity of a metabolomics fingerprint under cannabis as compared to a range of other drugs and performance measures. Finally, future research may explore whether treating distinctions in cannabis use patterns as a continuum rather than a binary variable may better capture the variability in metabolomic effects and behaviors associated with cannabis use.

In sum, the current study has demonstrated the feasibility of the metabolomics approach to identify metabolic changes that are specific to the neurocognitive state of cannabis intoxication and to the frequency of cannabis use. Such metabolomic fingerprints may provide more discriminative power to classify and separate cannabis use and cannabis impairment, than the mere detection of cannabinoids in blood.

## Supplementary information

Supplement

supplement

## Data Availability

The data of this study are available on reasonable request from the corresponding author, pending scientific review and a completed data use agreement.
