# Supplementary material for: Metabolomic profiling of cannabis use and cannabis intoxication in humans
Source: Neuropsychopharmacology. 2025 Mar 12;50(6):920–7. doi: 10.1038/s41386-025-02082-7 (PMC12032370; doi:10.1038/s41386-025-02082-7)
Supplement: Supplementary file 1 — Supplement [file 41386_2025_2082_MOESM1_ESM.docx]

**Supplement**

**Metabolomic profiling of cannabis use and cannabis intoxication in humans**

Francisco Madrid-Gambin^a^, Noemí Haro^a^, Natasha L. Mason^b^, Pablo Mallaroni^b^, Eef L Theunissen ^b^, Stefan W. Toennes ^c^, Oscar J. Pozo^a^. Johannes G. Ramaekers ^b^

^a^Applied Metabolomics Research Group, Hospital del Mar Research Institute, 08003 Barcelona, Spain

^b^Department of Neuropsychology and Psychopharmacology, Faculty of Psychology and Neuroscience, Maastricht University, 6200 MD, Maastricht, the Netherlands

^c^ Goethe University Frankfurt, University Hospital, Institute of Legal Medicine, Germany

 Correspondence: j.ramaekers@maastrichtuniversity.nl

**Table S1.** Descriptive characteristics

| **Demographics** | Occasional users  *N* = 18 | Chronic users  *N* = 17 | *P* value |
| --- | --- | --- | --- |
| Age (years)^1^ | 22.3 ± 3.32 | 21.8 ± 2.2 | 0.557 |
| Gender^2^  Male  Female | 7 (38.9%)  11 (61.1%) | 8 (47.1%)  9 (52.9%) | ​​​0.625 |
| Cannabis use per month | 3.3 ± 2.6 | 19.8 ± 10.4 | <0.001* |

^1^P-values from independent t-test between occasional and chronic users are shown for quantitative variables, presented as mean ± standard deviation. ^2^Pearson’s Chi-squared test is shown for the categorical variable.

**Table S2**. List of targeted biomarkers

| Family | Metabolite | Abbreviation |
| --- | --- | --- |
| Corticosteroids | Cortisol | F |
| Corticosteroids | 20α-Dihydrocortisol | DHF20a |
| Corticosteroids | 20β-Dihydrocortisol | DHF20b |
| Corticosteroids | 5α-Tetrahydrocortisol | THF5a |
| Corticosteroids | 5β-Tetrahydrocortisol | THF5b |
| Corticosteroids | 6-Hydroxycortisol | OH6F |
| Corticosteroids | Cortisone | E |
| Corticosteroids | 20α-Dihydrocortisone | DHE20a |
| Corticosteroids | 20β-Dihydrocortisone | DHE20b |
| Corticosteroids | Corticosterone | B |
| Corticosteroids | 11-Dehydrocorticosterone | A |
| Corticosteroids | 11-Deoxycorticosterone | DOC |
| Corticosteroids | Testosterone | T |
| Corticosteroids | Androstenedione | AED |
| Corticosteroids | 11β-dehidrogenase | 11bHSD |
| Corticosteroids | 20α/20β-Dihydrocortisol | 20α/20β-DHF |
| Corticosteroids | 6-Hydroxycortisol/Cortisol | 6OHF/F |
| Energy metabolism | Lactate | Lactate |
| Energy metabolism | Citrate | Citrate |
| Energy metabolism | Succinate | Succinate |
| Energy metabolism | Fumarate | Fumarate |
| Energy metabolism | Malate | Malate |
| Energy metabolism | Fumarate/Succinate | Fum/Succ |
| Energy metabolism | Malate/Fumarate | Mal/Fum |
| Energy metabolism | Citrate/Malate | Cit/Mal |
| Energy metabolism | 3-hydroxyButyrate | 3OHBut |
| Energy metabolism | 2-hydroxyGlutarate | 2OHGlut |
| Energy metabolism | 2-hydroxyButyrate | 2OHBut |
|  | Isovalerate | Isovalerate |
|  | Valerate | Valerate |
| Amino Acid metabolism | Valine | Valine |
| Amino Acid metabolism | Leucine | Leucine |
| Amino Acid metabolism | Isoleucine | Isoleucine |
| Amino Acid metabolism | Methionine | Methionine |
| Amino Acid metabolism | Hippurate | Hippurate |
| Amino Acid metabolism | Phenylalanine | Phenylalanine |
| Amino Acid metabolism | Tryptophan | Tryptophan |
| Amino Acid metabolism | Tyrosine | Tyrosine |
| Amino Acid metabolism | Glutamate | Glutamate |
| Amino Acid metabolism | Glutamine | Glutamine |
| Amino Acid metabolism | Serotonin | 5HT |
| Amino Acid metabolism | HIAA5 | HIAA5 |
| Amino Acid metabolism | Kynurenine | Kynurenine |
| Amino Acid metabolism | Kynurenate | Kynurenate |
| Amino Acid metabolism | Glutamate/Glutamine | Glu/Gln |
| Amino Acid metabolism | Valine/LNAA | Val/LNAA |
| Amino Acid metabolism | Leucine/LNAA | Leu/LNAA |
| Amino Acid metabolism | Isoleucine/LNAA | Ile/LNAA |
| Amino Acid metabolism | Tryptophan/LNAA | Trp/LNAA |
| Amino Acid metabolism | Phenylalanine/LNAA | Phe/LNAA |
| Amino Acid metabolism | Tyrosine/LNAA | Tyr/LNAA |
| Amino Acid metabolism | Serotonin/Tryptophan | Kyn/Trp |
| Amino Acid metabolism | Kynurenine/Tryptophan | X5HT/Trp |
| Amino Acid metabolism | Creatinine | Creatinine |
| Amino Acid metabolism | Creatine | Creatine |
| Acylglycerol | DAG 16:1 | DAG 16:1 |
| Acylglycerol | DAG 16:0 | DAG 16:0 |
| Acylglycerol | DAG 16:0 18:2 | DAG 16:0 18:2 |
| Acylglycerol | DAG 18:1 16:0 | DAG 18:1 16:0 |
| Acylglycerol | DAG 16:0 18:0 | DAG 16:0 18:0 |
| Acylglycerol | DAG 18:2 | DAG 18:2 |
| Acylglycerol | DAG 18:0 18:2 | DAG 18:0 18:2 |
| Acylglycerol | DAG 18:1 | DAG 18:1 |
| Acylglycerol | DAG 18:0 18:1 | DAG 18:0 18:1 |
| Acylglycerol | DAG 18:0 | DAG 18:0 |
| Acylglycerol | DAG 18:0 20:4 | DAG 18:0 20:4 |
| Acylglycerol | MAG 16:0 | MAG 16:0 |
| Acylglycerol | MAG/DAG 18:2 | MAG/DAG 18:2 |
| Acylglycerol | MAG/DAG 18:1 | MAG/DAG 18:1 |
| Acylglycerol | MAG/DAG 20:4 | MAG/DAG 20:4 |
| Lysophosphatidylcholine | LPC 16:0 | LPC 16:0 |
| Lysophosphatidylcholine | LPC 18:0 | LPC 18:0 |
| Ceramides | Cer 18:1 14:0 | Cer 18:1 14:0 |
| Ceramides | Cer 18:1 16:0 | Cer 18:1 16:0 |
| Ceramides | Cer 18:1 18:0 | Cer 18:1 18:0 |
| Ceramides | Cer 18:1 20:0 | Cer 18:1 20:0 |
| Ceramides | Cer 18:1 22:0 | Cer 18:1 22:0 |
| Ceramides | Cer 18:1 24:0 | Cer 18:1 24:0 |
| Ceramides | Cer 18:1 24:1 | Cer 18:1 24:1 |
| Ceramides | HexCer d18:1 16:0 | HexCer d18:1 16:0 |
| Ceramides | HexCer d18:1 18:0 | HexCer d18:1 18:0 |
| Ceramides | HexCer d18:1 22:0 | HexCer d18:1 22:0 |
| Ceramides | HexCer d18:1 24:0 | HexCer d18:1 24:0 |
| Ceramides | HexCer d18:1 18:1 | HexCer d18:1 18:1 |
| Ceramides | HexCer d18:1 24:1 | HexCer d18:1 24:1 |
| Ceramides | HexCer/Cer 16:0 | HexCer/Cer 16:0 |
| Ceramides | HexCer/Cer 18:0 | HexCer/Cer 18:0 |
| Ceramides | HexCer/Cer 22:0 | HexCer/Cer 22:0 |
| Ceramides | HexCer/Cer 24:0 | HexCer/Cer 24:0 |
| Ceramides | HexCer/Cer 24:1 | HexCer/Cer 24:1 |
| Sphingolipid | Sphingomyelin | Sphingomyelin |
| Sphingolipid | Sphingosine | Sphingosine |
|  | Choline | Choline |
|  | Acetylcholine | Acetylcholine |
|  | Acetylcarnitine | Acetylcarnitine |
|  | Carnitine | Carnitine |
|  | Adenosine | Adenosine |
|  | Trimethylamine N-oxide | TMAO |
|  | Betaine | Betaine |
|  | Trimethylamine N-oxide/Choline | TMAO/Choline |
|  | Acetylcholine/Choline | Ach/Chol |
| Endocannabinoids | 2-Arachidonoylglycerol | 2-AG |
| Endocannabinoids | 2-Linoleoylglycerol | 2-LG |
| Endocannabinoids | 2-Oleoylglycerol | 2-OG |
| Endocannabinoids | Anandamide (N-Arachidonoylethanolamine) | AEA |
| Endocannabinoids | Palmitoylethanolamide | PEA |
| Endocannabinoids | Oleoylethanolamide | OEA |
| Endocannabinoids | Palmitoleoylethanolamide | POEA |
| Endocannabinoids | Stearoylethanolamide | SEA |
| Endocannabinoids | Linoleoylethanolamide | LEA |
| Endocannabinoids | Docosahexaenoylethanolamide | DHEA |
| Endocannabinoids | Dihomo-γ-linolenoylethanolamide | DGLEA |
| Endocannabinoids | Docosatetraenoylethanolamide | DEA |

**Table S3.** Metabolic changes in occasional and chronic cannabis users after smoking cannabis.

| Cannabis  user group | Metabolite | Family | Slope^a^ | Wald^b^ | *P*-value^b^ | FDR |
| --- | --- | --- | --- | --- | --- | --- |
| Occasional | β-Hydroxybutyrate | ketone body | -0.821 | 7.44 | 0.006 | 0.033 |
|  | Glutaric acid | AA metabolism | -0.017 | 7.41 | 0.006 | 0.033 |
|  | Lactic acid | Energy metabolism | -4.080 | 6.44 | 0.011 | 0.033 |
|  | α-Hydroxybutyrate | Energy metabolism | -0.849 | 6.43 | 0.011 | 0.040 |
|  | α-Hydroxyglutarate | Citric acid cycle | -0.003 | 5.73 | 0.017 | 0.047 |
|  | Malic acid | Citric acid cycle | -0.046 | 5.14 | 0.023 | 0.047 |
|  | Succinic acid | Citric acid cycle | -0.033 | 4.87 | 0.027 | 0.047 |
|  | Isocitric acid | Citric acid cycle | -0.001 | 4.69 | 0.030 | 0.047 |
|  | Tryptophan | Tryptophan metabolism | -0.206 | 4.72 | 0.030 | 0.690 |
|  | Citric acid | Citric acid cycle | -0.369 | 4.35 | 0.037 | 0.051 |
|  | PEA | Endocannabinoid | 0.128 | 4.30 | 0.038 | 0.270 |
|  | DEA | Endocannabinoid | 0.009 | 3.88 | 0.049 | 0.270 |
|  |  |  |  |  |  |  |
| Chronic | Isoleucine | BCAA metabolism | -4.950 | 9.90 | 0.002 | 0.038 |
|  | Tyrosine | AAA metabolism | -3.917 | 8.13 | 0.004 | 0.039 |
|  | Leucine | BCAA metabolism | -3.281 | 7.87 | 0.005 | 0.039 |
|  | Cortexolone | Steroid | -0.026 | 4.95 | 0.026 | 0.313 |
|  | β-Hydroxybutyrate | ketone body | -0.599 | 4.88 | 0.027 | 0.298 |

^a^Slope in logarithm format * 1000. ^b^Wald score from GLMs as ratio of the estimated feature value to its standard error. ^c^P-value from Treatment by Time interaction. Abbreviations: AAA, aromatic amino acid; BCAA, branched chain amino acid; DEA, docosatetraenoylethanolamide; FDR, false discovery rate; PEA, N-palmitoylethanolamine.

**Table S4.** Metabolites associated with psychomotor vigilance task performance in occasional cannabis users

|  |  | Attention task 10 min after vaporization | | | Attention task 30 min after vaporization | | |
| --- | --- | --- | --- | --- | --- | --- | --- |
| Metabolite | Family | Beta | *P*-value | FDR | Beta | *P*-value | FDR |
| LPC 18.0 | LPC | 0.447 | 0.021 | 0.034 | 0.118 | 0.013 | 0.022 |
| LPC 16.0 | LPC | 1.128 | 0.023 | 0.034 | 0.243 | 0.015 | 0.022 |
| HexCer d18:1 24:0 | Ceramide | 0.737 | 0.005 | 0.028 | 0.155 | 0.022 | 0.046 |
| HexCer d18:1 22:0 | Ceramide | 0.642 | 0.013 | 0.04 | 0.151 | 0.02 | 0.046 |
| HexCer d18:1 24:1 | Ceramide | 0.495 | 0.108 | 0.217 | 0.17 | 0.023 | 0.046 |
| DAG 18:0 | Diacylglycerol | -0.386 | 0.614 | 0.844 | 0.152 | 0.008 | 0.087 |
| Cer 18:1 24:0 | Ceramide | 0.465 | 0.027 | 0.085 | 0.097 | 0.113 | 0.396 |
| Cer 18:1 24:1 | Ceramide | 0.773 | 0.036 | 0.085 | 0.183 | 0.083 | 0.396 |
| Cer 18:1 20:0 | Ceramide | 0.924 | 0.007 | 0.051 | 0.137 | 0.219 | 0.509 |
| SEA | Endocannabinoid | -3.046 | 0.011 | 0.122 | 0.453 | 0.174 | 0.661 |
| PEA | Endocannabinoid | -2.256 | 0.03 | 0.166 | 0.318 | 0.217 | 0.661 |

Abbreviations: LPC, lysophosphatidylcholine; PEA, N-palmitoylethanolamine; SEA, N-stearoylethanolamine

**Table S5.** Metabolites associated with psychomotor vigilance task performance in chronic cannabis users

|  |  | Attention task 10 min after vaporization | | | Attention task 30 min after vaporization | | |
| --- | --- | --- | --- | --- | --- | --- | --- |
| Metabolite | Family | Beta | P-value | FDR | Beta | P-value | FDR |
| 2-LG | Endocannabinoid | 0.454 | 0.001 | 0.009 | 0.195 | 0.444 | 0.869 |
| 20β-DHE | Steroid | 2.254 | 0.036 | 0.231 | 0.983 | 0.599 | 0.798 |
| 5α-THF | Steroid | 0.543 | 0.039 | 0.231 | 0.035 | 0.938 | 0.938 |
| α-Ketobutyrate | Citric acid cycle | -0.902 | 0.026 | 0.405 | -0.682 | 0.295 | 0.8 |
| DAG 18:0 20:4 | Diacylglycerol | -0.257 | 0.681 | 0.981 | -2.128 | 0.016 | 0.176 |
| Kynurenine | TRP metabolism | 0.503 | 0.694 | 0.976 | 4.48 | 0.003 | 0.074 |

Abbreviations: 2-LG, 2-linoleoyl glycerol; 20β-DHE, 20β-dihydrocortisone; 5α-THF, 5α-tetrahydrocortisol; DAG, diacylglycerol; TRP, tryptophan.

**Table S6.** Metabolites associated with ratings of subjective high in occasional cannabis users

|  |  | Subjective High 20 min after vaporization | | | Subjective High 40 min after vaporization | | |
| --- | --- | --- | --- | --- | --- | --- | --- |
| Metabolite | Family | Beta | *P*-value | FDR | Beta | *P*-value | FDR |
| Cer.18:1 20:0 | Ceramide | 0.579 | 0.018 | 0.123 | 0.077 | 0.827 | 0.931 |
| Cer.18:1 24:0 | Ceramide | 0.295 | 0.040 | 0.140 | 0.017 | 0.931 | 0.931 |
| Fumaric acid | Citric acid cycle | -0.170 | 0.684 | 0.999 | -1.170 | 0.009 | 0.149 |
| HexCer.d18:1 22:0 | Ceramide | 0.419 | 0.006 | 0.025 | -0.160 | 0.484 | 0.832 |
| HexCer.d18:1 24:0 | Ceramide | 0.414 | 0.008 | 0.025 | -0.219 | 0.339 | 0.832 |
| HMB | BCAA metabolism | -1.080 | 0.026 | 0.440 | -0.950 | 0.148 | 0.419 |
| LPC 16:0 | LPC | 0.536 | 0.035 | 0.035 | -0.532 | 0.114 | 0.228 |
| LPC 18:0 | LPC | 0.258 | 0.033 | 0.035 | -0.158 | 0.345 | 0.345 |

Abbreviations: BCAA, branched-chain amino acid; HexCer; hexosylceramide; HMB, β-Hydroxy β-methylbutyric acid; LPC, lysophosphatidylcholine

**Table S7.** Metabolites associated with ratings of subjective high in chronic cannabis users

|  |  | Subjective High 20 min after vaporization | | | Subjective High 40 min after vaporization | | |
| --- | --- | --- | --- | --- | --- | --- | --- |
| Metabolite | Family | Beta | *P*-value | FDR | Beta | *P*-value | FDR |
| Betaine | AA metabolism | -0.763 | 0.038 | 0.322 | -0.576 | 0.360 | 0.829 |
| DAG 16.0 | Diacylglycerol | 1.564 | 0.042 | 0.175 | 1.705 | 0.179 | 0.492 |
| DAG 16.0.18.0 | Diacylglycerol | 1.515 | 0.021 | 0.175 | 2.382 | 0.042 | 0.240 |
| DAG 18.0.18.1 | Diacylglycerol | 1.201 | 0.048 | 0.175 | 0.605 | 0.521 | 0.637 |
| Fumaric acid | Citric acid cycle | 0.014 | 0.949 | 0.982 | 0.800 | 0.008 | 0.138 |
| Lactic acid | Citric acid cycle | -0.038 | 0.915 | 0.982 | 1.158 | 0.026 | 0.224 |
| LPC 16.0 | LPC | -0.232 | 0.403 | 0.403 | -0.947 | 0.022 | 0.044 |
| Testosterone | Steroid | 0.694 | 0.040 | 0.475 | 0.016 | 0.979 | 0.979 |
| Valine | BCAA metabolism | -0.555 | 0.036 | 0.322 | -0.082 | 0.865 | 0.969 |

Abbreviations: AA, amino acid; DAG, diacylglycerol; LPC, lysophosphatidylcholine


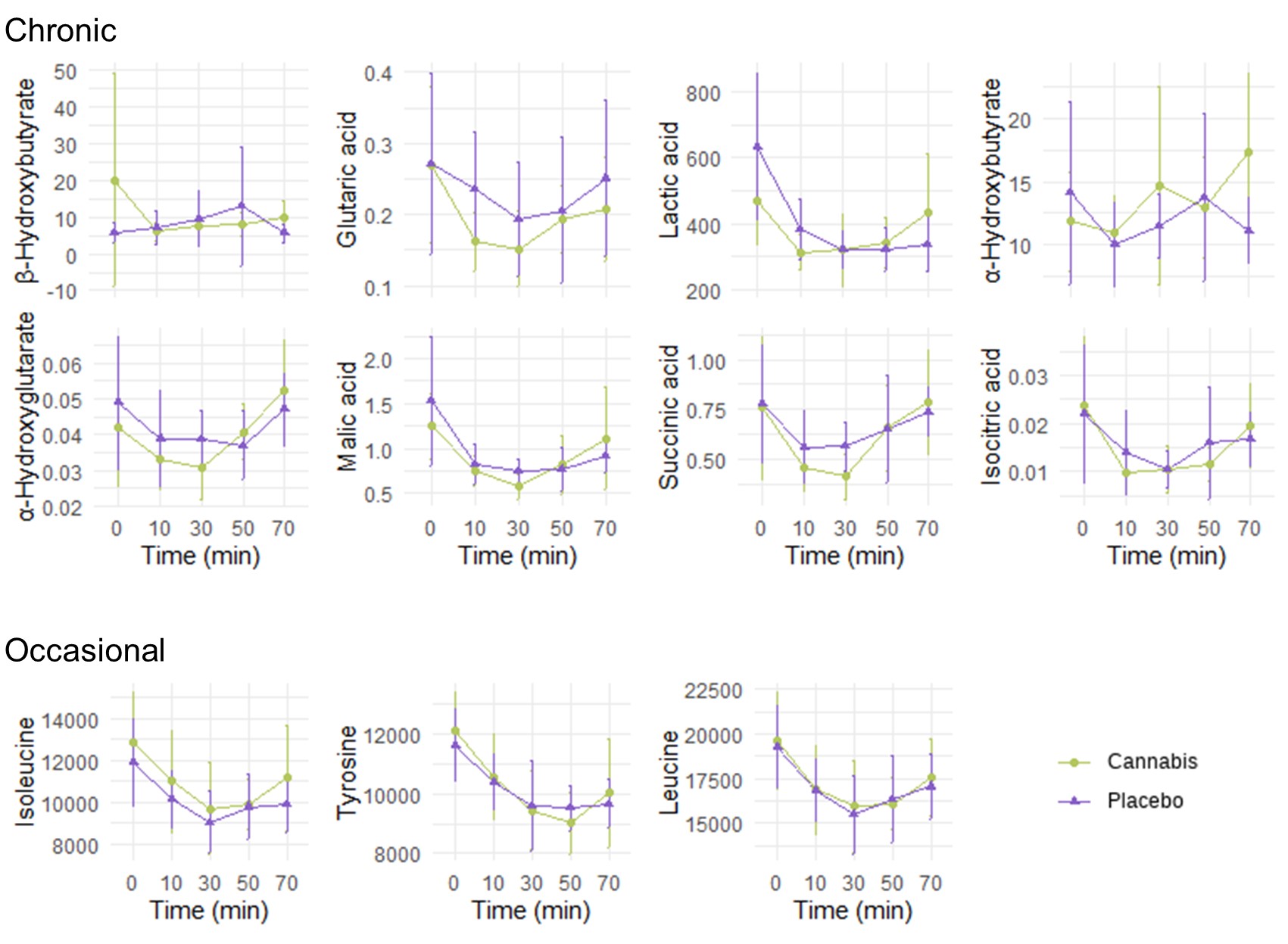


**Figure S1.** Kinetic profiles of non-significant, condition-specific metabolites complementary to Figure 2 of the main manuscript. The top panels display metabolites that showed significant changes over time in occasional cannabis users (Figure 2) but not in chronic users as shown here, while the bottom panel depicts metabolites that were significantly altered in chronic (Figure 2) but not in occasional users as shown here.
