# Supplementary figures and images for: Metabolomic profiling of cannabis use and cannabis intoxication in humans
Source: Neuropsychopharmacology. 2025 Mar 12;50(6):920–7. doi: 10.1038/s41386-025-02082-7 (PMC12032370; doi:10.1038/s41386-025-02082-7)

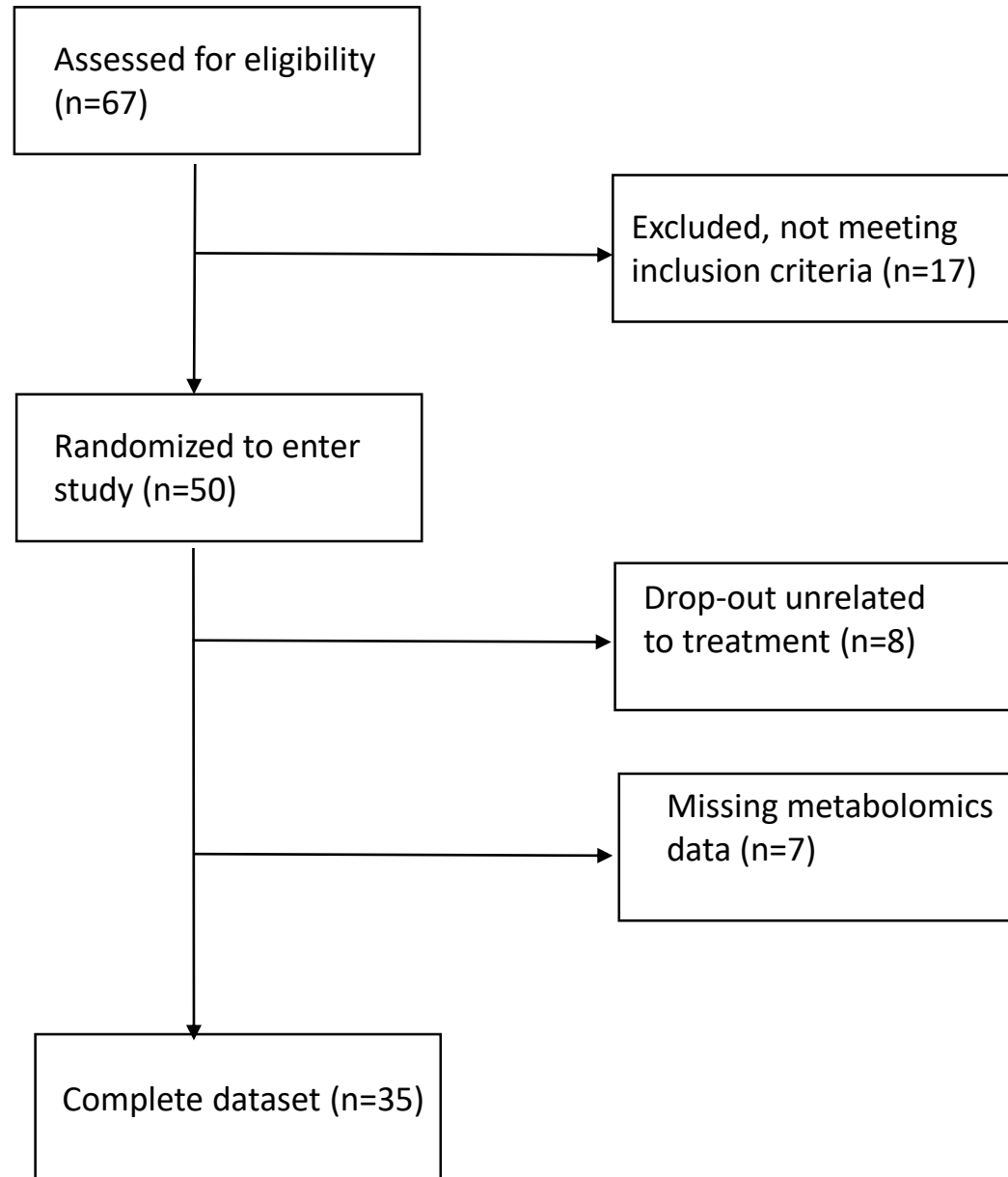

Supplement: Supplementary file 2 — supplement [file 41386_2025_2082_MOESM2_ESM.pdf]
